# Functional and Lateral Asymmetry of the Knee Joint Muscles Measured Using Tensiomyography (TMG) in Professional Football Players of Different Playing Positions

**DOI:** 10.3390/healthcare13010067

**Published:** 2025-01-02

**Authors:** Lazar Pajović, Lazar Toskić, Aleksandar Joksimović, Adem Preljević, Dina Joksimović, Slavko Dragosavljević, Djordje Stanić, Ljubiša Lilić, Veroljub Stanković, Borislav Cicović

**Affiliations:** 1 Serbian Institute of Sport and Sports Medicine, 11000 Belgrade, Serbia; lazar.pajovic@yahoo.com; 2 Faculty of Sport and Physical Education, University of Priština-Kosovska Mitrovica, 38218 Leposavić, Serbia; djordje.stanic@pr.ac.rs (D.S.); lilic.ljubisa@pr.ac.rs (L.L.); veroljub.stankovic@pr.ac.rs (V.S.); 3 Faculty of Sport, University “Union-Nikola Tesla”, 11000 Belgrade, Serbia; 4 Institute of Applied Technology, Abu Dhabi 20300, United Arab Emirates; alex.joksimovic@actvet.gov.ae (A.J.); dina.joksimovic@actvet.gov.ae (D.J.); 5 Biomedical Sciences Department, State University of Novi Pazar, 36300 Novi Pazar, Serbia; apreljevic@np.ac.rs; 6 Faculty of Physical Education and Sport, University of East Sarajevo, 71420 Pale, Bosnia and Herzegovina; slavko.dragosavljevic@ffvis.ues.rs.ba (S.D.); borislav.cicovic@ffvis.ues.rs.ba (B.C.)

**Keywords:** asymmetry, knee joint muscles, football players, playing positions

## Abstract

**Background/Objectives**: The aim of this study is to determine whether different playing positions in football influence muscle asymmetry, which is a common cause of injuries in football. This study aimed to determine the difference in the functional and lateral asymmetry of the knee joint muscles measured using tensiomyography (TMG) between football players of different playing positions. **Methods**: This study included 52 professional football players (25.1 ± 4.7 years) divided into three groups according to their playing position: defenders—DF (N = 18), midfielders—MF (N = 15), and forwards—FW (N = 19). Functional and lateral symmetry were investigated by applying TMG on the knee joint muscles Rectus femoris, Vastus medialis, Vastus lateralis, Biceps femoris, and Semitendinosus of the right and left leg, and using the algorithm proposed by the manufacturer and previous studies. **Results**: The results of one and two-way ANOVA revealed no significant differences in functional (F = 0.596, *p* = 0.555, on average) and lateral asymmetry (F = 0.888, *p* = 0.497, on average) from the aspect of muscle contraction velocity and muscle stiffness between football players of different playing positions. **Conclusions**: The study results show that the specificity of the playing position in football does not influence the functional and lateral asymmetry of the knee joint muscles. However, it has been shown that there is a high percentage of players with lateral (n = 14.5, 39.6%, on average) and specifically functional asymmetry (n = 27, 51.9%, on average).

## 1. Introduction

Symmetry, in general, may be defined as the quality of an object to demonstrate an exact correspondence of size, shape, and form across its two halves when split along a given axis, while asymmetry presents various types of deviation from symmetry [1]. In the human body functioning, muscle asymmetry, especially regarding lower limbs, plays an important role in everyday movement and sports activities. Lower limb muscle asymmetry can be classified as inter-limb (differences between the limbs) and intra-limb (differences within the same limb) asymmetry [2]. The dominance of one group of muscles may result in asymmetries, which can be understood as differences in strength, power, balance, flexibility, stiffness, electrical muscle activity, range of motion, etc. [3,4,5]. These differences are important factors in sports performance, the training process, and injury prevention [1,6,7].

Lower limb muscle asymmetry is an interesting phenomenon and is commonly investigated in sports where the muscle properties of the legs play a fundamental role in performance, such as football. Previous research shown that lower limb asymmetry can result in a decline in jump height, kicking velocity and accuracy, change in direction, speed time, etc. [1,5]. Since these movement patterns are one of the most important aspects of football performance, an appropriate functional relationship between the left and right leg muscles, and more importantly, the knee joint extensor/flexor muscles, is significant for successful performance [5,8,9]. More importantly, knee joint muscle asymmetry is a common cause of injury in football [10,11,12]. Potential mechanisms for injury caused by muscle asymmetry may include the limitation of an athlete’s movement strategies, which leads to performing tasks in an inefficient or dysfunctional manner, causing fatigue or micro-trauma to accumulate, which can further lead to injury [5]. It has been shown that hamstring muscle strain is the most common type of injury in football, influenced by muscle asymmetry [10,11,12].

There are numerous methods for the assessment of knee joint muscle asymmetry. It is mainly assessed through the differences between muscle groups in muscle strength and power, where isotonic (various jump types on the force plates) and isokinetic dynamometry represent the gold standard [13,14,15]. Also, lower limb asymmetry can be assessed through the differences in flexibility [16], electromyographic activity—EMG [17], etc.

A relatively new method for the assessment of muscle asymmetry is tensiomyography (TMG) which is based on the measurement of electrically induced muscle contractile properties [18,19,20]. Tensiomyography is frequently used for determining the lateral (inter-limb) and functional (intra-limb) asymmetry of the knee joint muscles in football [21,22,23]. However, only one of these studies investigated the differences in lower limb asymmetry measured by the TMG method between male football players of different playing positions [22], while apparently no studies investigated these differences in professional male football players.

Understanding the nature of players’ position characteristics in football can be of great value for sports training, injury prevention, the selection process, and the achievement of top-level results [24]. Previous studies revealed that each playing position in football has specific performance (covered distance, running intensity, acceleration, and deceleration, etc.) and technical demands [25,26,27]. Moreover, it has been shown that there are differences in injury patterns between football players of different playing positions [28], which muscle asymmetry may influence.

Following the aforementioned, the goal of this study is to assess whether different playing positions in football influence muscle asymmetry. This study aimed to determine the difference in the functional and lateral asymmetry of the knee joint muscles between football players of different playing positions, measured using tensiomyography (TMG). It is hypothesized that there will be significant differences in the muscle asymmetry values of knee joint muscles between football players who specialize in different playing positions.

## 2. Materials and Methods

### 2.1. Sample of Participants

This study included 52 professional football players (Age = 25.1 ± 4.7 years, body weight = 81.2 ± 6.8 kg, body height = 183.8 ± 5.2 cm) divided into three groups according to their playing position: defenders—DF (N = 18), midfielders—MF (N = 15), and forwards—FW (N = 19). The players were members of teams from the Serbian Super League (Novi Pazar, Radnički Niš, Radnički Kragujevac). The inclusion criteria for participating in the study were as follows: a minimum of 5 years of experience in professional football activity; a minimum of 10 years of practice experience; and active participation in competitions. The exclusion criteria were the presence of current injuries or illness. The study purpose and the protocol were explained to the participants, and each participant provided written consent for participation in the study. The study was conducted following the ethical standards of the Declaration of Helsinki and the rules of the Ethics Committee of the Faculty of Physical Education and Sport, University of East Sarajevo (protocol code 1628/22).

### 2.2. Procedures

Measurements of muscle contractile properties were conducted using the TMG method (TMG-BMC Ltd., Ljubljana, Slovenia). Measured muscles were knee extensors and flexor muscles of the right and left leg: Rectus femoris (RF), Vastus medialis (VM), Vastus lateralis (VL), Biceps femoris (BF), and Semitendinosus (ST).

The measurement procedure followed the manufacturer’s instructions and previous studies’ methodologies [24,29,30]. Participants were rested before the measurement. Then, measurements were performed under static, relaxed conditions, with the subject in the supine position for the measurements of RF, VM, and VL muscles, and in the prone position for BF and ST muscles. Electrodes (Pals Platinum, model 895220 with a multi-stick gel, Axelgaard Manufacturing Co., Ltd., Fallbrook, CA, USA) were positioned inside muscle borders (proximally and distally) and the sensor (GK40, Panoptik, Ljubljana, Slovenia) location was at the midpoint between both electrodes, at the center of the muscle belly. The electrical impulse of 1 ms duration was performed using an electrostimulator TMG-100 (TMG-BMC, Ljubljana, Slovenia), while the initial impulse was set at an intensity of 20 mA and then proportionally increased by 10 mA steps until the maximal value was achieved, namely until the moment when any muscle reaction to the increase in the electrostimulation ceased. The two best scores were used for further statistical analysis. The tensiomyography parameters used in this study were contraction time (Tc—time between 10% and 90% of the maximum value of the muscle response), delay time (Td—the time required to reach 10% of the maximum value of the muscle response), relaxation time (Tr—the time taken to lower the contraction from 90 to 50% of the maximum value of the muscle response), maximal displacement (Dm—maximal radial displacement of muscle belly), and sustain time (Ts—the time passed from 50% at the stage of the contraction up to 50% at the relaxation phase). The experienced staff conducted all measurements at the end of the competition season.

The TMG software algorithm was used to calculate the functional and lateral symmetries as follows [20,22,23]:

$$\begin{array}{l} \mathrm{Functional}\;\mathrm{symmetry}:\;\mathrm{FS}=0.1\times (\frac{(MIN(AVERAGE\left(TdRF;TdVL;\;TdVM\right):TdBF}{(MAX(AVERAGE\left(TdRF;TdVL;\;TdVM\right):TdBF})+0.8\times (\frac{(MIN(AVERAGE\left(TcRF;TcVL;\;TcVM\right):TcBF}{(MAX(AVERAGE\left(TcRF;TcVL;\;TcVM\right):TcBF})+0.1\times \\ (\frac{(MIN(AVERAGE\left(TrRF;TrVL;\;TrVM\right):TrBF}{(MAX(AVERAGE\left(TrRF;TrVL;\;TrVM\right):TrBF})\times 100. \end{array}$$

$$\mathrm{Lateral}\;\mathrm{symmetry}:\;\mathrm{LS}=0.1\times (\frac{\left(MIN(TdR;TdL\right)}{MAX\left(TdR;TdL\right)})+0.6\times (\frac{\left(MIN(TcR;TcL\right)}{MAX\left(TcR;TcL\right)})+0.1\times (\frac{\left(MIN(TsR;TsL\right)}{MAX\left(TsR;TsL\right)})+0.2\times (\frac{\left(MIN(DmR;DmL\right)}{MAX\left(DmR;DmL\right)})\times 100.$$

Additionally, lateral asymmetry was determined by investigating the differences between groups in individual TMG parameters.

### 2.3. Statistical Analysis

The statistical procedures used in the study included descriptive statistics (Mean, SD), and univariate analyses of variance (ANOVA), while the distribution of normality was calculated by the Kolmogorov–Smirnov (KS) test. One-way ANOVA was applied to determine the differences in the functional and lateral asymmetry between football players of different playing positions using the algorithm proposed both by the manufacturer and in previous studies. Two-way ANOVA was applied to investigate the lateral asymmetry between groups in individual TMG parameters (leg × position interaction). Effect sizes were calculated using partial eta squared (η^2^) and interpreted as small (0.01), moderate (0.06), or large (0.14) [31]. The Bonferroni post hoc test was applied for the parameters that showed statistical significance. The statistical significance level was 95% with *p* < 0.05. All the statistical procedures were performed using the SPSS19 (IBM) program, IBM Armonk, New York, NY, USA.

## 3. Results

Table 1 and Table 2 present the results of descriptive statistics of measured TMG parameters of the right and left knee joint extensor and flexor muscles in football players of different playing positions, as well as the results of the two-way ANOVA, that is, the interaction between differences in individual TMG parameters of the right and left leg (lateral asymmetry) and players’ position. It can be observed that there are no significant differences in lateral asymmetry (defined by the differences between the right and left leg in individual TMG parameters) between football players of different playing positions regarding knee joint extensors (F = 0.817, *p* = 0.551, η^2^ = 0.017, on average) and flexors muscles (F = 0.642, *p* = 0.654, η^2^ = 0.016, on average).

**Figure 1:**
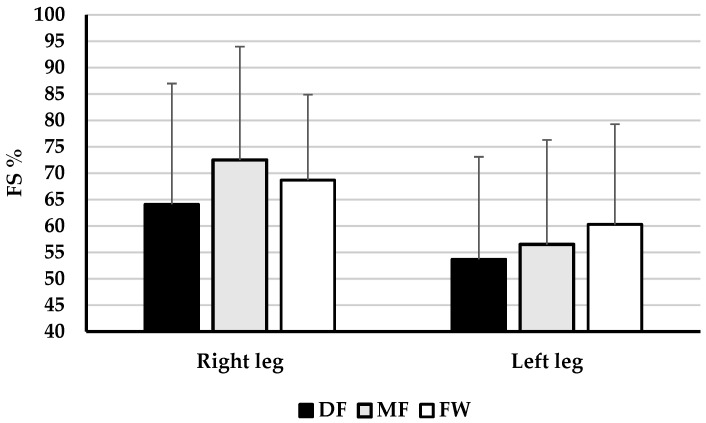
Descriptive statistics (Mean ± SD) of functional asymmetry of right and left leg and the ANOVA results—differences between football players of different playing positions.

**Figure 2:**
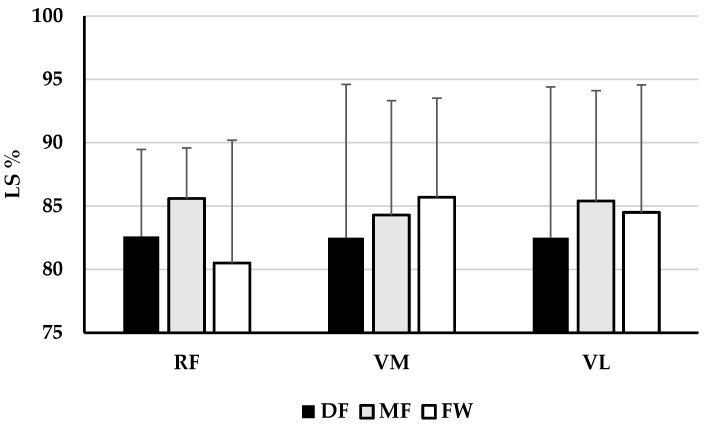
Descriptive statistics (Mean ± SD) of lateral asymmetry of knee joint extensor muscles and the ANOVA results—differences between football players of different playing positions.

**Figure 3:**
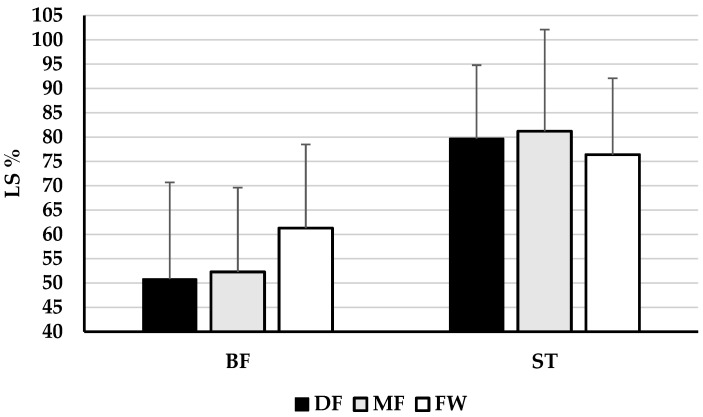
Descriptive statistics (Mean ± SD) of lateral asymmetry of knee joint flexor muscles and the ANOVA results—differences between football players of different playing positions.

Figure 1, Figure 2 and Figure 3 present the descriptive results of functional asymmetry of the right and left leg, and lateral symmetry of the knee joint extensor and flexor muscles in football players of different playing positions, as well as the results of one-way ANOVA, that is, differences in asymmetry between groups. It has been shown that there are no significant differences in the functional asymmetry of the right (F = 0.618, *p* = 0.543, η^2^ = 0.023) and left leg (F = 0.574, *p* = 0.568, η^2^ = 0.024) between football players of different playing positions. Also, it can be concluded that there are no significant differences in the lateral asymmetry of muscles RF (F = 1.614, *p* = 0.205, η^2^ = 0.067), VM (F = 0.446, *p* = 0.643, η^2^ = 0.019), VL (F = 0.310, *p* = 0.735, η^2^ = 0.013), BF (F = 1.737, *p* = 0.187, η^2^ = 0.070), and ST (F = 0.335, *p* = 0.717, η^2^ = 0.014) between football players of different playing positions.

## 4. Discussion

This study investigates the functional and lateral asymmetry of knee joint muscles in professional football players of different playing positions. To the best of the authors’ knowledge, this is the first study to investigate this problem in the field. The results could contribute to developing the training process, talent identification, and injury prevention in football.

The main finding of this study is that there are no significant differences in the functional and lateral asymmetry of the knee joint muscles between football players of different playing positions. Namely, the observed results revealed no significant differences in the functional asymmetry of the right and left leg (F = 0.596, *p* = 0.555, η^2^ = 0.023, on average, Figure 1) and lateral asymmetry of knee joint extensor and flexor muscles RF, VM, VL, BF and ST (F = 0.888, *p* = 0.497, η^2^ = 0.036 on average, Figure 2 and Figure 3). Additionally, the results showed no significant differences in lateral asymmetry investigated through individual TMG parameters between groups (F = 0.817, *p* = 0.551, η^2^ = 0.017, on average, Table 1 and Table 2).

These results are in accordance with the results of previous similar studies. Paravlic et al. [23] investigated the inter-limb differences in lower limb muscles measured by the TMG method between female football players of different playing positions. The study results showed no significant differences between groups in functional (F = 1.383, *p* = 0.259) and lateral asymmetry (F = 1.140, *p* = 0.439, on average). Additionally, a study by Buoite Stella et al. [22], which investigated the muscle asymmetries in lower limb muscles measured by the TMG method of sub-elite male football players, revealed that there are no significant differences in the lateral asymmetry of muscles RF, VM, VL, and BF between football players according to playing positions, while there are differences in the functional asymmetry (F = 5.683, *p* = 0.012) of the knee joint muscles with players in central roles presenting significantly worse symmetry scores than defense players, especially in the non-dominant limb.

As previously mentioned, lateral (inter-limb) asymmetry is defined as a difference between the limbs, while functional (intra-limb) asymmetry represents a difference within the same limb [2]. In the case of TMG method and knee joint muscles, lateral asymmetry represents the differences in parameters Td, Tc, Ts, and Dm between measured muscles of the right and left leg. The functional asymmetry is defined as a difference in Td, Tc, and Tr between knee joint extensors muscle RF, VM, and VL and flexor muscle BF [20,22,23]. TMG parameters mainly refer to various muscle contraction velocity properties (Tc, Td, Tr) and muscle stiffness (Dm), where parameters Tc and Dm have shown the highest validity [18,19,30]. Accordingly, the present study has shown no differences in functional and lateral asymmetry from the aspect of the muscle contraction velocity and stiffness of the knee joint muscles between football players of different playing positions. Namely, there are no differences between DF, MF, and FW players. These results are expected to a certain extent, since previous studies are inconsistent regarding the influence of playing position on knee joint muscle contractile properties, and some of them revealed no significant differences [24,32,33]. The observed results indicate that the specificity of the playing position in football (movement patterns, energy consumption, etc.) does not influence the functional and lateral asymmetry of the knee joint muscles. Also, it can be assumed that the training process is equally implemented in all players, regardless of their playing position.

However, functional and lateral asymmetry in the included subjects of football players is present, revealing interesting information. The cut-off values for lateral and functional asymmetry are 80% and 65%, respectively [20,22,23]. The observed results showed that, regardless of the playing position, 40.3% of players have functional asymmetry (values lower than 65%) for the right leg and 63.4% for the left leg. These numbers are lower when it comes to lateral asymmetry, with 25% of players having below 80% cut-off value for the RF muscle, 21.1% for the VM, 28.8% for the VL, 36.5% for the ST, and the highest percentage of football player with lateral asymmetry is found for the BF muscle, being 86.5%. When playing position is considered, there are 50% of DF players, 26.6% of MF players and 42.1% of FW players with functional asymmetry of the right leg, while there are 66.6% of DF players, 60% of MF players and 63.1% of FW players regarding left leg. Similarly to whole group observation, the number of players with lateral asymmetry is lower when considering the playing position, with an average of 30.5% for DF players, 21.6% for MF players, and 30.2% for FW players regarding the RF, VM, VL, and ST muscles. Again, the highest percentage of players with lateral asymmetry is shown to be with BF muscle, where 88.8% of DF players, 86.6% of MF players, and 84.2% of FW players have lateral asymmetry.

It can be concluded from previously stated information that in professional football players, functional asymmetry (n = 27, 51.9%, on average) of the knee joint muscles is more frequent than lateral asymmetry (n = 14.5, 39.6%, on average). Also, functional asymmetry can occur more often regarding the left rather than the right leg (for 23.1%). Further, it has been shown that the highest percentage of subjects have lateral asymmetry in the BF muscle (86.5%). Finally, it can be concluded that DF players, although there are no statistically significant differences between groups, more frequently have functional and lateral asymmetry of the knee joint muscles (46.8%, on average) than MF (37.1%, on average) and FW (44.3%, on average) players. These high percentages of football players with detected functional and lateral asymmetry point to caution. Although lateral asymmetry is not strongly associated with performance or injury risk in sports [34], a disturbed functional relationship between the knee joint flexor and extensor muscles can lead to injury [6,31,35]. Interestingly, functional asymmetry is higher in the left leg, which most participants stated was a non-dominant leg. These results indicate that, although the dominant leg is more sensitive to injuries [10,11,12,36,37], the training process in football should equally be focused on the flexor/extensor relationship of both legs. Also, the BF muscle once more confirmed its role as a muscle which is highly sensitive to injuries in football [38,39]. Finally, it can be assumed that these high levels of asymmetry are caused by the competition period when study data were collected, since the measurements were conducted at the end of the competing period.

This is one of the study’s limitations. The present study has investigated functional and lateral asymmetry in football players at the end of the competing period, which is important since the level of fatigue and injury frequency is high at this point in time [35,40,41]. However, future studies should focus on the investigation of this problem during the season. Another study limitation is the sample of participants. Namely, this study did not include the goalkeepers, since there are very few available professional goalkeepers for participating in this kind of study. It would be important and interesting to reveal the functional and lateral knee joint asymmetry in these players and compare them to football players of other playing positions. Finally, players’ injury history was not considered, which could influence the results.

## 5. Conclusions

The main finding of this study is that there are no significant differences in the functional and lateral asymmetry of knee joint muscles measured using the TMG method between professional football players of different playing positions. Namely, there are no differences in functional and lateral asymmetry from the aspect of the muscle contraction velocity and stiffness of the knee joint muscles. The observed results show that the specificity of the playing position in football does not influence the functional and lateral asymmetry of the knee joint muscles. However, it has been shown that there is a high percentage of players with lateral and especially functional asymmetry. The results of this study indicate that the training process in football, which is focused on overall performance and prevention of injuries caused by muscle asymmetry, should be equally implemented in all players, regardless of playing position.

## Figures and Tables

**Table 1:** Descriptive statistics (Mean ± SD) of measured TMG parameters of the knee extensors muscles in football players of different playing positions and the results of two-way ANOVA.

			Defenders	Midfielders	Forwards	ANOVA		
						F	*p*	η^2^
**Rectus femoris**	**Tc (ms)**	**Right leg**	31.4 ± 6.64	29.2 ± 4.62	32.4 ± 8.14	0.416	0.661	0.009
		**Left leg**	28.9 ± 5.77	28.6 ± 4.83	28.7 ± 6.41			
	**Ts (ms)**	**Right leg**	152.2 ± 86.1	128.3 ± 46.8	128 ± 38.5	1.502	0.228	0.032
		**Left leg**	106.9 ± 42.3	103.9 ± 43.8	124.1 ± 29.2			
	**Tr (ms)**	**Right leg**	76.4 ± 29.1	67.6 ± 33	70.6 ± 33.2	2.387	0.098	0.049
		**Left leg**	58.5 ± 28.8	56 ± 25.6	81.3 ± 24.9			
	**Dm (mm)**	**Right leg**	6.26 ± 2.99	6.05 ± 1.66	6.94 ± 2.52	0.223	0.800	0.005
		**Left leg**	5.57 ± 2.32	6.02 ± 2.25	6.74 ± 2.16			
	**Td (ms)**	**Right leg**	25.6 ± 5.02	23.4 ± 1.94	24.4 ± 2.83	1.628	0.202	0.034
		**Left leg**	23.6 ± 2.20	24 ± 2.28	24.6 ± 2.83			
**Vastus medialis**	**Tc (ms)**	**Right leg**	28.7 ± 6.70	28.8 ± 6.63	27.2 ± 4.26	1.574	0.213	0.033
		**Left leg**	31.9 ± 10.8	28.5 ± 6.63	24.8 ± 3.93			
	**Ts (ms)**	**Right leg**	174.2 ± 24.3	173.2 ± 26.4	172.2 ± 18.4	0.009	0.991	0.000
		**Left leg**	179.5 ± 30.3	178.2 ± 63.5	175.3 ± 45.9			
	**Tr (ms)**	**Right leg**	45.4 ± 19.8	36.5 ± 7.03	42 ± 13.6	0.077	0.926	0.002
		**Left leg**	47.4 ± 19.1	41.0 ± 19.5	48.1 ± 38.1			
	**Dm (mm)**	**Right leg**	6.38 ± 1.73	5.64 ± 1.32	5.44 ± 2.12	0.190	0.828	0.004
		**Left leg**	5.96 ± 1.92	5.75 ± 1.71	5.47 ± 1.94			
	**Td (ms)**	**Right leg**	22.3 ± 1.50	22 ± 1.65	22 ± 1.74	1.656	0.197	0.035
		**Left leg**	24.4 ± 2.39	22.8 ± 1.52	22.6 ± 2.16			
**Vastus lateralis**	**Tc (ms)**	**Right leg**	27.1 ± 6.23	26.2 ± 6.16	24.5 ± 3.73	0.647	0.526	0.014
		**Left leg**	25.8 ± 4.13	24.7 ± 4.68	25.4 ± 3.56			
	**Ts (ms)**	**Right leg**	128.5 ± 22.7	120.7 ± 37.2	119.9 ± 35.7	0.146	0.864	0.003
		**Left leg**	125.9 ± 22.5	124.8 ± 37.2	124.4 ± 28.7			
	**Tr (ms)**	**Right leg**	60.1 ± 13.3	54.5 ± 16.2	61.8 ± 27.9	1.094	0.339	0.023
		**Left leg**	70.7 ± 26.3	63.9 ± 30	58.3 ± 15.2			
	**Dm (mm)**	**Right leg**	4.23 ± 1.78	3.93 ± 1.15	3.83 ± 1.47	0.373	0.690	0.008
		**Left leg**	3.99 ± 1.60	4.13 ± 1.06	4.16 ± 1.52			
	**Td (ms)**	**Right leg**	24.4 ± 3.98	22.9 ± 1.83	22.4 ± 1.43	0.342	0.711	0.007
		**Left leg**	27 ± 14.1	23.9 ± 3.24	22.5 ± 1.95			

Legend: Tc—Contraction time, Ts—Sustain time, Tr—Relaxation time, Dm—Maximal displacement, Td—Delay time.

**Table 2:** Descriptive statistics of measured TMG parameters of the knee flexors muscles in football players of different playing positions and the results of two-way ANOVA.

			Defenders	Midfielders	Forwards	ANOVA		
						F	*p*	η^2^
**Biceps femoris**	**Tc (ms)**	**Right leg**	20.2 ± 8.86	27.7 ± 16.6	23.1 ± 11.3	1.029	0.362	0.022
		**Left leg**	20.6 ± 14.7	18.5 ± 13.4	20.8 ± 13.3			
	**Ts (ms)**	**Right leg**	137.3 ± 88.4	142.4 ± 75.8	155.2 ± 86.1	0.228	0.796	0.005
		**Left leg**	110.8 ± 73.6	91.5 ± 58.6	128.8 ± 69.5			
	**Tr (ms)**	**Right leg**	43.5 ± 33.7	52.9 ± 32.8	37.7 ± 19.4	3.066	0.060	0.089
		**Left leg**	29.04 ± 29.5	16 ± 17.2	52 ± 44.3			
	**Dm (mm)**	**Right leg**	1.71 ± 1.51	2.47 ± 1.64	2.63 ± 1.67	0.178	0.837	0.004
		**Left leg**	1.38 ± 2.38	1.48 ± 2.43	2.03 ± 2.26			
	**Td (ms)**	**Right leg**	26.4 ± 26.3	19.8 ± 5.76	20.5 ± 2.94	0.786	0.459	0.017
		**Left leg**	21.4 ± 10.08	20.7 ± 9	24.2 ± 17.9			
**Semitendinosus**	**Tc (ms)**	**Right leg**	46.6 ± 13.04	45.8 ± 14.5	43.9 ± 13.3	0.106	0.899	0.002
		**Left leg**	47.9 ± 13.9	43.8 ± 17.6	44.7 ± 14.6			
	**Ts (ms)**	**Right leg**	154.1 ± 59.2	157.9 ± 33.5	145.7 ± 38	0.378	0.686	0.008
		**Left leg**	152.6 ± 37.3	137.6 ± 45.9	139.4 ± 30			
	**Tr (ms)**	**Right leg**	66.2 ± 45.3	50.4 ± 20.6	55.9 ± 29	0.183	0.833	0.004
		**Left leg**	58.9 ± 20.5	43.5 ± 16.8	41.9 ± 13.2			
	**Dm (mm)**	**Right leg**	5.02 ± 3.08	5.74 ± 2.82	4.95 ± 2.42	0.447	0.641	0.010
		**Left leg**	6.25 ± 2.84	5.64 ± 3.37	5.41 ± 2.84			
	**Td (ms)**	**Right leg**	26.7 ± 7.70	25.1 ± 4.21	24.3 ± 4.62	0.026	0.974	0.001
		**Left leg**	26.4 ± 3.41	25 ± 4.21	23.6 ± 3.74			

Legend: Tc—Contraction time, Ts—Sustain time, Tr—Relaxation time, Dm—Maximal displacement, Td—Delay time.

## Data Availability

The data are available on request to the corresponding author.
